# Two Decades of Female Breast Cancer Mortality in Hungary: Epidemiological Trends Since EU Accession

**DOI:** 10.3390/cancers17244034

**Published:** 2025-12-18

**Authors:** Tamás Lantos, Tibor András Nyári, Giuseppe Verlato

**Affiliations:** 1 Department of Medical Physics and Informatics, Albert Szent-Györgyi Medical School, University of Szeged, 6720 Szeged, Hungary; lantos.tamas@med.u-szeged.hu; 2 Unit of Epidemiology and Medical Statistics, Department of Diagnostics and Public Health, University of Verona, 37134 Verona, Italy; giuseppe.verlato@univr.it

**Keywords:** breast cancer, female cancer deaths, cancer epidemiology, declining trend, regional differences, childless mortality, seasonality, Hungary, population-based study, COVID-19 pandemic

## Abstract

Objective: This study aimed to investigate annual and seasonal trends, as well as regional differences, in female breast cancer mortality in Hungary between 2004 and 2023. Methods: Data on cancer mortality were obtained from the publicly available nationwide population register. Poisson and quasi-Poisson regression models were applied to investigate the annual trend in breast cancer mortality rates. Cyclic trends in mortality were analysed using the Walter–Elwood method, and regional differences in age-standardised mortality rates (ASMRs) were evaluated across Hungarian regions. Results: Over the two decades studied, a total of 42,779 deaths from breast cancer were recorded. A significant declining trend in annual ASMRs for female breast cancer was observed during the study period (IRR = 0.996; 95% CI [0.993–0.998]; *p* = 0.002). Seasonal analysis revealed a significant cyclic pattern, with the highest number of deaths occurring during the winter months, peaking in December. The highest age-standardised breast cancer mortality rate (43.9 ± 0.2 per 100,000 female persons per year) was observed in the Capital region (Budapest), while the lowest ASMR (36.2 ± 0.25 per 100,000 female persons per year) was found in the Northern Great Plain region (*p* = 0.028). Conclusions: Although Hungary has implemented a free national breast cancer screening programme, this study demonstrates that breast cancer mortality remains high in the country. Additionally, breast cancer mortality exhibits significant regional and seasonal variation. These findings underscore the need for targeted public health interventions and optimised resource allocation to improve outcomes.

## Simple Summary

This study demonstrates that breast cancer mortality remains high in Hungary and exhibits significant regional and seasonal variation. The territorial proportions of age-standardised breast cancer deaths varied significantly, ranging from 36.16 to 43.8 deaths per 100,000 women in the study period. The lowest and highest mortality rates were observed in regions of the Northern Great Plain and Budapest (capital), respectively. Nevertheless, mortality from breast cancer in childless women was highest in the Budapest region. Additionally, a significant single peak seasonal pattern was detected in the proportionate mortality rates of breast cancer, with a peak in August and a nadir in February. These findings underscore the need for targeted public health interventions and optimised resource allocation to improve outcomes.

## 1. Introduction

In 2022, breast cancer was the second most common type of cancer globally among women [1]. Moreover, Hungary had the highest number of female breast cancer deaths in the European Union in 2012 and 2017 [2], and it remains the highest [3].

Breast cancer is one of the most common malignancies among Hungarian women. In Hungary, approximately 8000 new cases of breast cancer are diagnosed annually [4], and the regional differences in female breast cancer mortality have not yet been investigated. Furthermore, the breast cancer mortality rate is high in Hungary, as a large proportion of cases are diagnosed at a later stage [5]. Additionally, Hungary has higher rates of overweight and obesity, low physical activity, insufficient fruit and vegetable consumption, occupational exposures, air pollution, and daily smoking compared to other EU countries [2,4].

This ecological study aimed to assess regional differences, as well as annual and cyclic trends, in female breast cancer mortality in Hungary over the 20 years from 1 January 2004 to 31 December 2023. In addition, female breast cancer during the four years of the COVID-19 pandemic was compared with the non-pandemic period.

## 2. Materials and Methods

### 2.1. Study Population

This analysis covered the 20 years from 2004 to 2023. Data on the female population and breast cancer deaths were obtained from the published nationwide population register of the Hungarian Central Statistical Office (HCSO) [6]. Mortality data were classified under ICD-10 (International Classification of Diseases, 10th Revision) code C50. Data on both the population and cancer deaths were classified by the following age groups: 0–14, 15–39, 40–44, 45–49, 50–54, 55–59, 60–64, 65–69, 70–74, 75–79, and 80 years and older.

Similarly, monthly data on female breast cancer deaths were obtained from the HCSO; however, corresponding monthly data on the female population were not available. Therefore, annual female population data were extrapolated to estimate the population by month. The effects of education and the number of children as potential risk factors for female breast cancer were also investigated.

Monthly female breast cancer mortality rates were calculated as the number of deaths divided by the female population in the same month of the year. The proportionate breast cancer mortality rates were also calculated as the fraction of all female deaths attributable to this specific cause. Data on the month of death were aggregated over the study period. These aggregated monthly data were then used to calculate the relative change [(maximum − mean)/mean] to quantify the severity of seasonality and to compare seasonal amplitudes. This metric indicates the extent to which the maximum number of cases exceeds the mean value.

Territorial units were defined according to the NUTS 2016 classification (Nomenclature des unités territoriales statistiques—Nomenclature of Territorial Units for Statistics, 2016 revision) at the second level [7]. The statistical regions at the second level (NUTS2) were as follows: Budapest (the Capital region, formerly part of Central Hungary; HU11), Pest County (the surrounding region, formerly part of Central Hungary; HU12), Central Transdanubia (HU21), Western Transdanubia (HU22), Southern Transdanubia (HU23), Northern Hungary (HU31), Northern Great Plain (HU32), and Southern Great Plain (HU33).

### 2.2. Statistical Methods

Mortality rates were expressed per 100,000 females per year, using the annual mid-year female population estimates for the relevant year. Age-standardised mortality rates (ASMRs) in Hungary during the study period were calculated using the direct method [8] and the Revised European Standard Population (RESP) [9] to allow regional mortality rates to be comparable over time.

Data on the month of death were aggregated over the study period, and cyclic trends were analysed using the Walter–Elwood methods and Poisson regression [10,11]. Both methods account for the population at risk by grouping the data by month and were used to investigate the seasonal patterns. In contrast, the geometrical model developed by Edwards [12], which does not account for population size, was used as a sensitivity analysis to assess the impact of population effects on the cyclical trend.

Trends in the annual number of deaths were also analysed using Poisson and quasi-Poisson regression methods. Incidence rate ratios (IRRs) were calculated along with their corresponding 95% confidence intervals (95% CIs). Additionally, the non-pandemic period (2004–2019) was compared with the four years following the onset of the COVID-19 pandemic (2020–2023). A *p*-value < 0.05 was considered statistically significant. All analyses were performed using Stata Statistical Software: Release 17 (StataCorp LLC, College Station, TX, USA).

## 3. Results

Overall, 1,331,684 female deaths were registered in Hungary during the period 2004–2023, of which 42,779 were from breast cancer (Table 1). Most deaths (5895) from breast cancer occurred in the 70–74 year age group (Table 1). The crude mortality rate for breast cancer in the female population was 32.07 per 100,000 persons per year. After direct age-standardisation, the mortality rate for the female population only was 38.8 (SD [standard deviation] = 0.036) per 100,000 persons. However, the ASMR for breast cancer in the total Hungarian population was 23.4 (SD = 0.013) per 100,000 persons per year.

The highest age-specific mortality rates were observed among those aged over 85 years. The ASMRs increased in the older age groups for female breast cancer (IRR = 1.2; 95% CI [1.07–1.39]; *p* = 0.003).

The level of education is inversely associated with mortality rates from breast cancer (IRR = 0.94; 95% CI [0.93–0.95]; *p* < 0.001). In other words, individuals with higher levels of education had lower mortality rates. The proportion of breast cancer deaths was significantly higher among childless women than among women who had given birth (IRR = 0.96; 95% CI [0.95–0.97]; *p* < 0.001).

### 3.1. Annual Trends in Mortality

**Figure 1:**
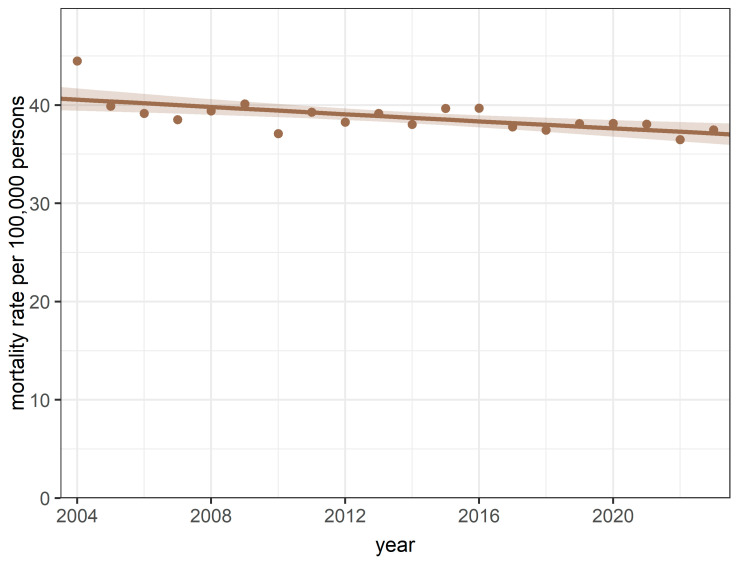
Annual trends in mortality rates during the study period between 2004 and 2023.

Annual age-standardised mortality rates for breast cancer ranged from 44.49 (SD = 0.90) per 100,000 female persons per year (2004) and 36.47 (SD = 0.64) per 100,000 female persons per year (2022) were observed (Figure 1).

The quasi-Poisson regression model for annual age-standardised data revealed a declining trend in the yearly ASMRs for breast cancer (IRR = 0.996; 95% CI [0.993–0.998]; *p* = 0.002 for trend and *p* = 0.16 for goodness-of-fit, respectively) during the study period. There was no significant difference in mortality rates between the COVID-19 and pre-COVID-19 periods (*p* = 0.83).

### 3.2. Seasonal Trends in Mortality

**Figure 2:**
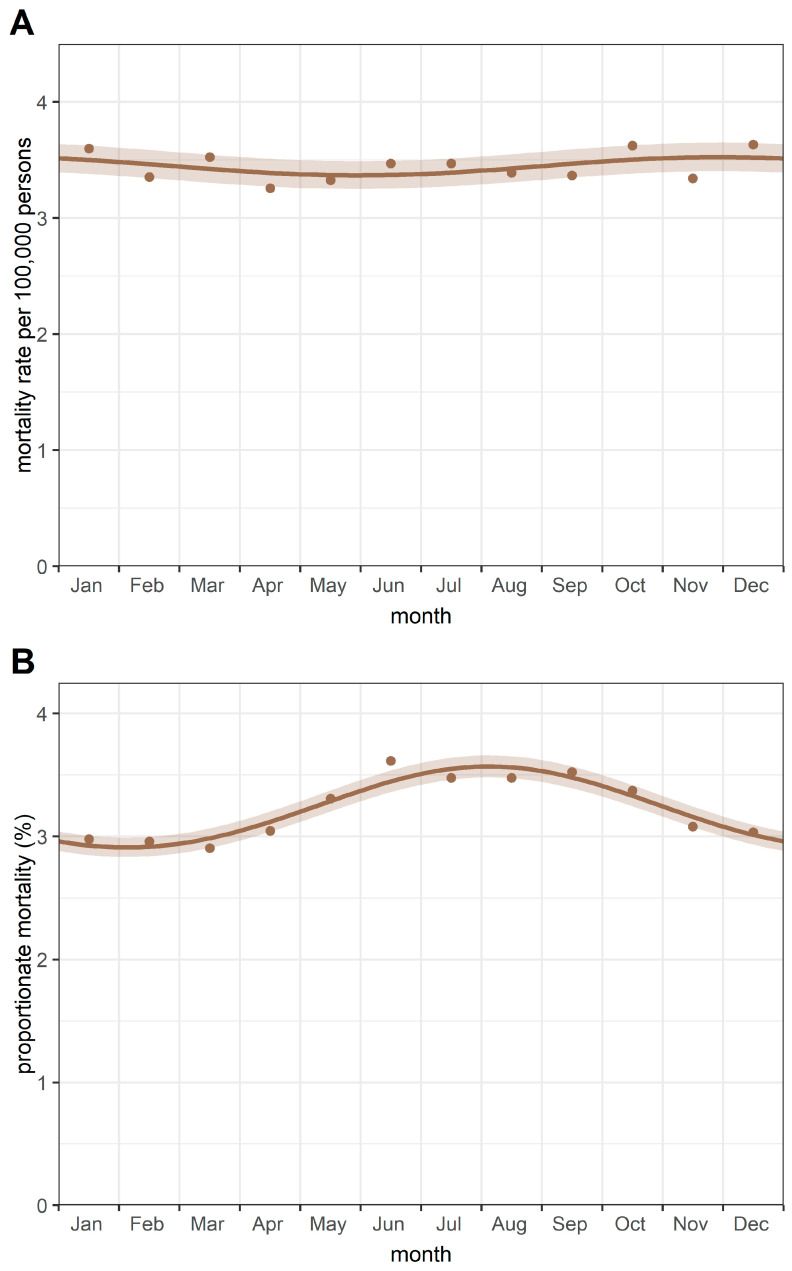
Cyclic trends of (**A**) mortality rates and (**B**) proportionate mortality from breast cancer. Notes: Mortality rates were calculated as deaths from female breast cancer divided by the female population (per 100,000 persons). Proportionate mortality was calculated as deaths from female breast cancer divided by all female deaths (expressed as a percentage).

The monthly aggregate of deaths is summarised in Table 2. Using the Walter–Elwood method, which accounted for the female population at risk, a significant (*p* = 0.004) single-peak cyclic trend was detected in monthly breast cancer deaths, with a peak in December (Figure 2A). However, the relative change of 2.23% suggests that the amplitude of the cyclic variation is small.

Additionally, a significant single peak seasonal pattern was detected in the proportionate mortality rates of breast cancer—using all female deaths as the underlying population—with a peak in August and a nadir in February (Figure 2B). A relative change of 5.1% was observed in proportionate mortality from breast cancer.

The Poisson regression method revealed seasonal trends similar to those identified by the Walter–Elwood method. Moreover, the application of the Edwards test confirmed the seasonality observed in the data.

### 3.3. Regional Differences

The regional distribution of female breast cancer mortality is summarised in Table 3.

**Figure 3:**
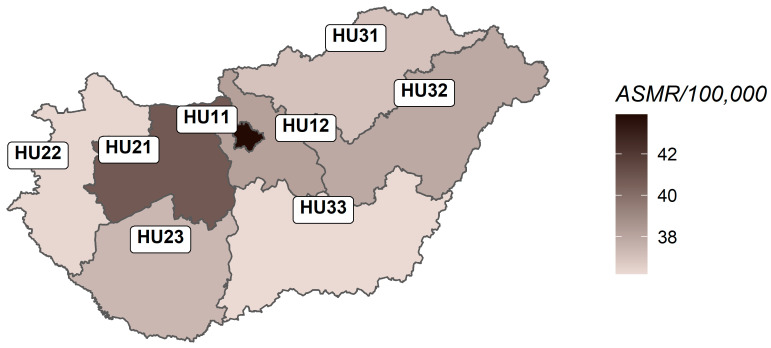
Age-standardised regional mortality rates from female breast cancer in Hungary, 2004–2024.

The territorial proportions of age-standardised breast cancer deaths varied significantly (*p* = 0.028), ranging from 36.16 to 43.8 deaths per 100,000 women in the study period (Table 3). The lowest and highest mortality rates were observed in regions of the Northern Great Plain (HU33) and Budapest (HU11, capital), respectively. Mortality rates were also high in Central Transdanubia (HU21) and Central Hungary (HU12) (Figure 3). Nevertheless, breast cancer mortality in childless women was highest in the Budapest region (16.8%).

## 4. Discussion

### 4.1. Main Findings

A significant declining trend in the yearly ASMRs for breast cancer was detected during the twenty-year study period. Most deaths occurred in winter, and using the observed numbers, a significant seasonal pattern in breast cancer deaths was revealed, with a peak in December. A significant inverse association was found between education level and female breast cancer deaths.

The highest age-standardised breast cancer mortality rate (ASMR) was observed in the Capital (Budapest), while the lowest ASMR was found in the Northern Great Plain region. Additionally, mortality from breast cancer in childless women was highest in the Budapest region.

### 4.2. Strengths and Weaknesses of Our Study

Mortality data were obtained from Hungary’s vital register, which is recognised as one of the highest-quality systems globally, reporting a vital statistics performance index of 95.7% close to the maximum value of 100% [13]. Although we had no individual data, our study confirmed that education and breastfeeding may be associated with breast cancer risk.

Our ecological study demonstrated significant regional and seasonal differences in the pattern of breast cancer mortality in Hungary. Despite the lack of direct evidence for respiratory syncytial virus infections in the analyses, we can speculate that these infections may play a role in the aetiology of breast cancer mortality. In addition, the main limitation of this study is the lack of data (e.g., genetic background of the patients, menopausal status, family history of breast cancer, and other important determinants), which would help in investigating and explaining the decrease in the mortality rate.

To the best of our knowledge, this is the first epidemiological study to report territorial differences in mortality for female breast cancer in Hungary.

### 4.3. Comparison with Other Studies

International breast cancer trends have varied over the last few decades, with many high-income countries showing declines in female breast cancer mortality despite stable or increasing incidence rates, whereas both mortality and incidence have increased in many lower- and middle-income countries. Similarly to Austria and Poland, breast cancer is the second most diagnosed malignant disease and the leading cause of cancer death in women in Hungary [14,15]. Although the ASMRs for breast cancer are decreasing, they are still higher than those reported for EU27 countries [16]. The COVID-19 pandemic has not led to an increase in mortality rates from female breast cancer in Hungary.

In the UK, cancer mortality in women aged 35–69 years was substantially reduced between 1993 and 2018 [17,18]. This decline is likely a reflection of the successes in cancer prevention, earlier detection, improved diagnostics, and more effective treatments. In a cohort study, Jiang et al. reported that higher education levels were associated with a significantly increased incidence of breast cancer [19]. We did not have access to data on female breast cancer incidence. However, breast cancer mortality rates were significantly higher among women with lower education levels compared to those with higher education levels. A similar inverse relationship between mortality and education was reported by Gotink et al. [20].

A study on breastfeeding found that it is associated with a reduced risk of breast cancer, with the risk decreasing by approximately 4.3% for every 12 months of breastfeeding [21]. Our study revealed that the breast cancer mortality rate is significantly higher in childless women. In a meta-analysis, Islami et al. reported that ever breastfeeding had a protective effect against hormone receptor-negative breast cancers, which generally have a poorer prognosis than other breast cancer subtypes [22].

Debella et al. reported in a recent meta-analysis that age over 40 years, family history, overweight, smoking, oral contraceptive use, air pollution, and night shift work were associated with increased odds of breast cancer. Furthermore, sufficient consumption of fruits and vegetables was linked to lower breast cancer risk, indicating a protective effect in their study [23].

Since 1990, the incidence of female breast cancer has been rising globally. Regional differences exist in the burden of female cancers related to socioeconomic development [5,24,25]. Although the age-standardised national breast cancer mortality rate in Romania slightly decreased between 2000 and 2020, rates increased in several regions [26]. Breast cancer mortality rates declined more in urban areas than in rural areas. However, breast cancer mortality remained higher in urban areas than in rural areas [26]. Age-standardised mortality rates exhibit considerable variations across Italian provinces, with higher breast cancer mortality in the northwest and lower rates in southern Italy [27]. Similarly, our study found the highest age-standardised breast cancer mortality rates in the Capital, Central Hungary, and Western Hungarian regions, which are the developed regions of the country.

Earlier studies by Nakaji et al. [28], Virág and Nyári [29], and Hartai et al. [30] reported significant seasonality in proportionate cancer mortality. Like previous findings, we observed significant seasonality in proportionate cancer mortality, with a summer peak. The number of all female deaths was used as the reference population to characterise the proportional mortality rates of breast cancer. However, analyses based on all female populations at risk revealed significant winter peak seasonality in breast cancer mortality rates. The observed inverse seasonal cycle appears to stem from the same underlying factor, exerting a considerably stronger effect on non-cancer mortality than on cancer mortality. This may partly explain the higher breast cancer mortality at the end of summer [29]. In this study, the relative change of 2.23% indicates that the amplitude of the cyclic variation is small. The decreased number of deaths in summer may be partly attributable to the minimal impact of respiratory conditions during this season. Additionally, nosocomial infections could increase the risk of mortality [31,32]. However, both seasonal patterns provide valuable insights for strengthening prevention efforts. In addition, investigating seasonality is an important component in understanding the aetiology of certain diseases. These findings, together with the observed regional disparities, suggest that environmental exposures, socioeconomic inequalities, and differences in healthcare access may influence mortality rates.

In Hungary, organised breast screening has been provided every 2 years for women aged 45 to 65 since 2002 [4,33]. Women can do the most to detect breast cancer and precancerous conditions as early as possible by participating in public health screening programmes.

Sardini et al. recently reported that mammography screening may reduce breast cancer mortality in the general population; however, efforts are needed to ensure optimal participation rates [34]. Giorgi et al. [35] summarised the results of breast cancer screening programmes conducted in Italy. Preliminary data showed a coverage rate of 69.4%, with 51.1% of the target population having received an invitation to screening. Although participation in organised breast cancer screening programmes is considered a primary prevention method [36,37,38]. Belicza et al. reported that participation rates in female breast cancer screening in Hungary declined between 2013 and 2020, with significant variation across counties. In addition, every year the participation rate was 5–7% higher in the 63–64 years age group than in the 45–46-year-old one [39]. Women with higher education levels are 50% more likely to attend breast cancer screening [3].

## 5. Conclusions

Despite the presence of biologically aggressive subtypes, breast cancer remains largely preventable as a cause of cancer mortality. Our ecological study described the seasonal pattern of female breast cancer mortality in Hungary, showing a peak during the winter months. These findings suggest that environmental exposures may influence mortality rates.

Although a free national breast cancer screening programme is available, our findings suggest that its uptake remains suboptimal. Public health interventions should aim to increase participation rates, particularly among higher-risk populations, including childless and less-educated women, who are currently underrepresented in screening programmes. Implementing tailored outreach strategies, community-based education, and measures to reduce structural barriers to screening could enhance healthcare equity and result in markedly lower breast cancer mortality nationwide. We also recommend that public health strategies encourage healthy lifestyles and focus on high-risk groups through early screening. Healthcare planners should consider the seasonality of different diseases in allocating resources. For instance, health professionals involved in respiratory or infectious diseases could concentrate on vacations during summer, while oncologists and other health workers involved in cancer care must face a sustained disease burden during the hot season. Nevertheless, low participation rates in female breast cancer screening in Hungary may, in part, result from anomalies in coding practices [39].

Further research is needed to investigate the underlying causes of seasonal variation in mortality and to assess the effectiveness of targeted screening and prevention programmes. Such measures may improve early detection and ultimately lessen the burden of breast cancer mortality in Hungary.

## Figures and Tables

**Table 1:** Crude and age-standardised mortality rates from female breast cancer, 2004–2024.

Age (Years)	Number of Deaths from Breast Cancer (C50)	Age-Specific Rate	ASMR (Female Population Only)	All Female Deaths	Female Population
0–14	0	0.00	0	5510	14,081,407
15–39	905	2.87	0.89	13,873	31,576,595
40–44	957	13.33	0.93	10,653	7,176,660
45–49	1795	25.62	1.79	19,844	7,005,151
50–54	2746	39.21	2.74	34,715	7,003,267
55–59	3637	52.04	3.38	52,520	6,988,734
60–64	4707	68.19	4.09	77,214	6,902,312
65–69	5453	84.39	4.64	105,118	6,461,396
70–74	5895	107.39	5.37	138,542	5,489,579
75–79	5973	133.54	5.34	195,894	4,472,875
80–84	5289	164.70	4.12	258,089	3,211,377
≥85	5422	221.63	5.54	419,712	2,446,377
Total	42,779		38.84	1,331,684	102,815,730

**Table 2:** The aggregated monthly numbers of all female deaths and the number of deaths from female breast cancer, 2004–2024.

Month	Number of Deaths from Breast Cancer (C50)	All Female Deaths	Mortality Rate (per 100,000 Women)	Female Population
1	3728	125,120	3.60	103,615,297
2	3473	117,336	3.35	103,593,341
3	3650	125,648	3.52	103,571,378
4	3373	110,734	3.26	103,527,455
5	3443	104,090	3.33	103,483,531
6	3591	99,318	3.47	103,483,531
7	3588	103,216	3.47	103,439,612
8	3505	100,793	3.39	103,395,686
9	3481	98,785	3.37	103,351,767
10	3743	110,984	3.62	103,329,807
11	3452	112,054	3.34	103,307,843
12	3752	123,638	3.63	103,263,917
Total	42,779	1,331,716	3.45	1,241,363,165

**Table 3:** Age-standardised regional numbers of all female deaths and the number of deaths from female breast cancer, 2004–2024.

Regions	Number of Deaths from Breast Cancer (C50)	ASMR (C50)	SD	Number of Female Deaths	Proportion of Deaths from Breast Cancer in Childless Women
HU11	9170	43.85	0.21	384,129	16.8
HU12	4629	38.19	0.32	261,899	10.2
HU21	4744	40.85	0.36	262,399	10.6
HU22	3995	36.29	0.33	235,407	11.1
HU23	3981	37.40	0.35	239,697	9.5
HU31	4955	37.00	0.28	320,452	10.5
HU32	5855	37.89	0.25	353,424	11.6
HU33	5381	36.16	0.25	333,358	12.5

## Data Availability

Data are available in a public, open access repository. The data used in this study are available from: https://statinfo.ksh.hu/Statinfo/themeSelector.jsp?&lang=en (accessed on 15 December 2025).

## Abbreviations

The following abbreviations are used in this manuscript:

ASMR	Age-standardised mortality rate
CI	Confidence interval
HCSO	Hungarian Central Statistical Office
ICD-10	International Classification of Diseases, 10th Revision
IRR	Incidence rate ratio
NUTS	Nomenclature of Territorial Units for Statistics
RESP	Revised European Standard Population
SD	Standard deviation
